# Supplementary material for: Muscle transcriptome analysis reveals molecular pathways and biomarkers involved in extreme ultimate pH and meat defect occurrence in chicken
Source: Sci Rep. 2017 Jul 25;7:6447. doi: 10.1038/s41598-017-06511-6 (PMC5526995; doi:10.1038/s41598-017-06511-6)
Supplement: Supplementary file 1 — Supplementary Information [file 41598_2017_6511_MOESM1_ESM.pdf]

Muscle transcriptome analysis reveals molecular pathways and biomarkers involved in extreme ultimate pH and meat defect occurrence in chicken

Stéphane Beauclercq[1], Christelle Hennequet-Antier[1], Christophe Praud[1], Estelle Godet[1], Anne Collin[1], Sophie Tesseraud[1], Sonia Métayer-Coustard[1], Marie Bourin[2], Marco Moroldo[3], Frédéric Martins[4,5], Sandrine Lagarrigue[6], Elisabeth Le Bihan-Duval[1], Cécile Berri[*,1]

[1]URA, INRA, F-37380 Nouzilly, France

[2]ITAVI – Institut Technique de l'Aviculture, F-37380 Nouzilly, France

[3]GABI, AgroParisTech, INRA, Université Paris-Saclay, F-78350, Jouy-en-Josas, France

[4] Plateforme Genome et Transcriptome, Genopole de Toulouse, France

[5]INSERM, UMR1048, F-31432, Toulouse, France

[6]PEGASE, Agrocampus Ouest, INRA, F-35590, Saint-Gilles, France

[*]Corresponding author: cecile.berri@inra.fr

Gene Symbol	Description	Fold-change	UniProtKB, GO functional annotation
**Down-regulated genes**		**pHu-/pHu+**	
LSMEM1	leucine Rich Single-Pass Membrane Protein 1	0.21	
MTRF1	mitochondrial translational release factor 1	0.24	Mitochondrial translation release factor activity
CRH	corticoliberin precursor	0.28	synaptic transmission, dopaminergic
DOPEY1	dopey family member 1	0.36	golgi to endosome transport
RGCC	regulator of cell cycle	0.38	modulates the activity of cell cycle-specific kinases
MAPKAPK3	mitogen-activated protein kinase-activated protein kinase 3	0.41	activation of MAPK activity
ELFN1	extracellular leucine-rich repeat and fibronectin type III domain containing 1	0.42	postsynaptic protein with protein phosphatase inhibitor activity
SHISA2	protein shisa-2 homolog precursor	0.45	role in the maturation of presomitic mesoderm cells
CHRNG	acetylcholine receptor subunit gamma precursor	0.45	muscle-type acetylcholine receptor
CD79B	B-cell antigen receptor complex-associated protein beta chain precursor	0.46	adaptive immune response
**Up-regulated genes**		**pHu-/pHu+**	
KIAA1731	centrosomal Protein 295	3.92	mediates centriole-to-centrosome conversion at late mitosis
SCARNA23	small Cajal body-specific RNA 23	3.34	
CA9	carbonic anhydrase 9	3.12	one-carbon metabolic process
SLC25A30	solute carrier family 25 member 30	2.58	mitochondrial transport
FHL2	four and a half LIM domains 2	2.57	transcriptional regulation, focal adhesion
PGPEP1L	pyroglutamyl-peptidase I-like	2.41	cysteine-type peptidase activity
GADL1	glutamate decarboxylase like 1	2.39	may catalyze the decarboxylation of aspartate, cysteine sulfinic acid, and cysteic acid to beta-alanine, hypotaurine and taurine, respectively
FSTL4	follistatin-related protein 4 precursor	2.33	negative regulation of collateral sprouting
SLC34A2	Sodium-dependent phosphate transport protein 2B	2.30	sodium:phosphate symporter activity
KY	kyphoscoliosis peptidase	2.23	probable cytoskeleton-associated protease required for normal muscle growth

Supplementary Table S1. Top 10 up- and down-regulated genes between the pHu- and pHu+ (Only protein coding genes were included in this list)

Gene[a]	loading1	loaging2	loading3	loading4	loading5	loading6	loading7	loading8	loading9	loading10	Freq.[b]
ATP2B1.s2	0.123	0.235	0.248	0.193	0.135	0.208	0.151	0.258	0.111	0.176	1
LSMEM1.s2	0.525	0.400	0.377	0.393	0.349	0.425	0.406	0.372	0.382	0.412	1
CDAN1.s1	-0.052	-0.132	-0.118	-0.208	-0.061	-0.066	-0.077	-0.168	-0.040	-0.075	1
LOC100859584	0.115	0.206	0.075	0.148	0.162	0.124	0.120	0.154	0.255	0.133	1
LOC107052650	0.496	0.482	0.486	0.473	0.437	0.474	0.481	0.446	0.468	0.485	1
ID3.s1	0.164	0.089	0.137	0.072	0.036	0.093	0.075	0.192	0.070	0.080	1
MORN4.s1	0.113	0.162	0.112	0.218	0.218	0.175	0.133	0.165	0.216	0.219	1
MYLIP.s1	-0.068	-0.102	-0.037	-0.038	-0.136	-0.092	-0.037	-0.028	-0.048	-0.067	1
PCGF5.s1	0.036	0.093	0.073	0.096	0.190	0.067	0.060	0.083	0.170	0.097	1
PPFIBP1.s1	0.073	0.216	0.071	0.124	0.010	0.176	0.116	0.134	0.116	0.106	1
PRKCH.s1	-0.158	-0.188	-0.218	-0.183	-0.133	-0.184	-0.220	-0.213	-0.271	-0.241	1
SLC34A2	-0.079	-0.101	-0.124	-0.122	-0.129	-0.110	-0.112	-0.113	-0.025	-0.125	1
THSD7B.s2	-0.108	-0.111	-0.173	-0.113	-0.087	-0.105	-0.083	-0.116	-0.237	-0.168	1
TNS1.s2	0.026	0.089	0.176	0.111	0.073	0.065	0.043	0.065	0.072	0.058	1
CA9	-0.026	-0.025	-0.043	-0.049	NA	-0.056	-0.130	-0.024	-0.100	-0.011	0.9
CR353278	0.048	0.113	NA	0.079	0.181	0.057	0.099	0.119	0.013	0.155	0.9
DYNC1H1.s1	0.129	0.115	0.045	0.195	NA	0.130	0.153	0.277	0.123	0.137	0.9
ENC1.s2	-0.060	-0.147	-0.049	-0.085	-0.049	-0.072	-0.005	-0.012	NA	-0.070	0.9
GYLTL1B.s3	0.098	0.131	NA	0.089	0.231	0.099	0.032	0.095	0.057	0.099	0.9
HLF.s2	0.072	0.058	0.069	0.155	0.111	0.078	0.037	0.002	NA	0.136	0.9
PLEKHC1.s1	0.005	0.091	NA	0.061	0.055	0.097	0.130	0.077	0.092	0.018	0.9
LOC107049762	-0.182	-0.058	NA	-0.103	-0.057	-0.044	-0.004	-0.005	-0.030	-0.125	0.9
COG5.s2	0.039	0.079	0.025	0.040	0.022	0.339	0.020	0.095	NA	NA	0.8
CR338879	-0.067	-0.123	NA	-0.062	-0.057	-0.013	-0.033	-0.072	NA	-0.041	0.8
LOC768393	-0.026	-0.069	NA	-0.064	-0.055	-0.062	NA	-0.016	-0.017	-0.018	0.8
RHOC.s1	0.158	0.178	NA	0.182	0.131	NA	0.141	0.202	0.231	0.195	0.8
SLC7A5.s2	-0.033	-0.048	-0.170	-0.090	NA	-0.058	-0.024	-0.087	NA	-0.053	0.8
HECTD2.s1	NA	0.038	0.015	0.019	NA	0.025	0.007	0.113	0.076	0.046	0.8
ANKRD1.s2	0.060	0.028	NA	0.085	0.001	NA	NA	0.009	NA	0.009	0.6
VTI1B.s1	-0.262	-0.318	-0.323	NA	NA	-0.297	-0.317	NA	NA	-0.289	0.6
AMPD3.s1	NA	0.005	NA	0.092	NA	0.022	0.060	0.100	NA	0.067	0.6
BICD2.s2	NA	-0.032	-0.016	-0.052	NA	-0.008	-0.071	NA	NA	-0.034	0.6
RAI14.s2	NA	0.089	NA	0.012	NA	0.120	0.162	0.002	NA	0.021	0.6
MEF2C.s2	-0.095	-0.151	NA	-0.173	NA	-0.095	-0.073	NA	NA	NA	0.5
SEPT-09.s1	NA	0.060	NA	0.055	NA	NA	0.069	0.042	0.033	NA	0.5
CR406450	NA	NA	NA	0.012	0.125	0.029	NA	NA	0.104	0.095	0.5
CR386528	0.005	NA	NA	0.011	0.218	NA	NA	0.058	NA	NA	0.4
ZC2HC1A.s1	NA	0.066	NA	NA	NA	0.022	NA	0.021	NA	0.022	0.4
PFN2.s1	NA	NA	0.001	NA	NA	NA	NA	0.075	0.166	0.116	0.4
CYB5R3.s1	NA	NA	NA	0.036	NA	0.008	NA	NA	0.166	0.135	0.4

Supplementary table S2. Loadings of the 1st component of fsPLS models. The loading of genes or representative probes in the 10 fsPLS models performed at each CV step are tabled. The stability of gene selection in the models is indicated in Freq. column.

[a]The number following '.s' correspond to the reference of the representative probe. This information was used to target the primer design for quantitative RT-PCR validation. The genes highlighted in orange were part of the validation gene set.

[b]A cutoff was set at 40% for the stability of gene selection.

Gene	comp1	comp2	comp3
PRKCH	-0.2099	-0.3860	0.2314
THSD7B	-0.1975	-0.4033	0.3664
CA9	-0.1470	-0.5411	0.1925
ENC1	-0.1194	-0.3173	0.0408
SLC25A30	-0.1105	-0.3000	0.5930
VTI1B	-0.0207	-0.5964	0.1878
CAV3	0.2018	-0.2645	0.2758
PPFIBP1	0.2144	-0.1305	0.2101
SHISA2	0.2218	-0.0468	-0.2178
AMPD3	0.2231	-0.0657	0.2627
SLC2A1	0.2340	-0.1106	0.2219
PRKAB1	0.2435	-0.2423	-0.0476
ANKRD1	0.2451	-0.0966	0.0976
CR387500	0.2611	-0.1245	0.4526
MORN4	0.2787	-0.3347	-0.0310
RGCC	0.2792	-0.1500	-0.2529
MAPKAPK3	0.2907	-0.1237	-0.1367
PLEKHC1	0.2931	-0.2861	0.1777
PCGF5	0.2932	-0.0550	0.1186
RHOC	0.3189	-0.2368	-0.1188

Supplementary table S3. Loadings of the 3 components of the 20 genes kept in the final PLS model fitted on gene expression measured using microfluidic technology from the population of 278 chickens.

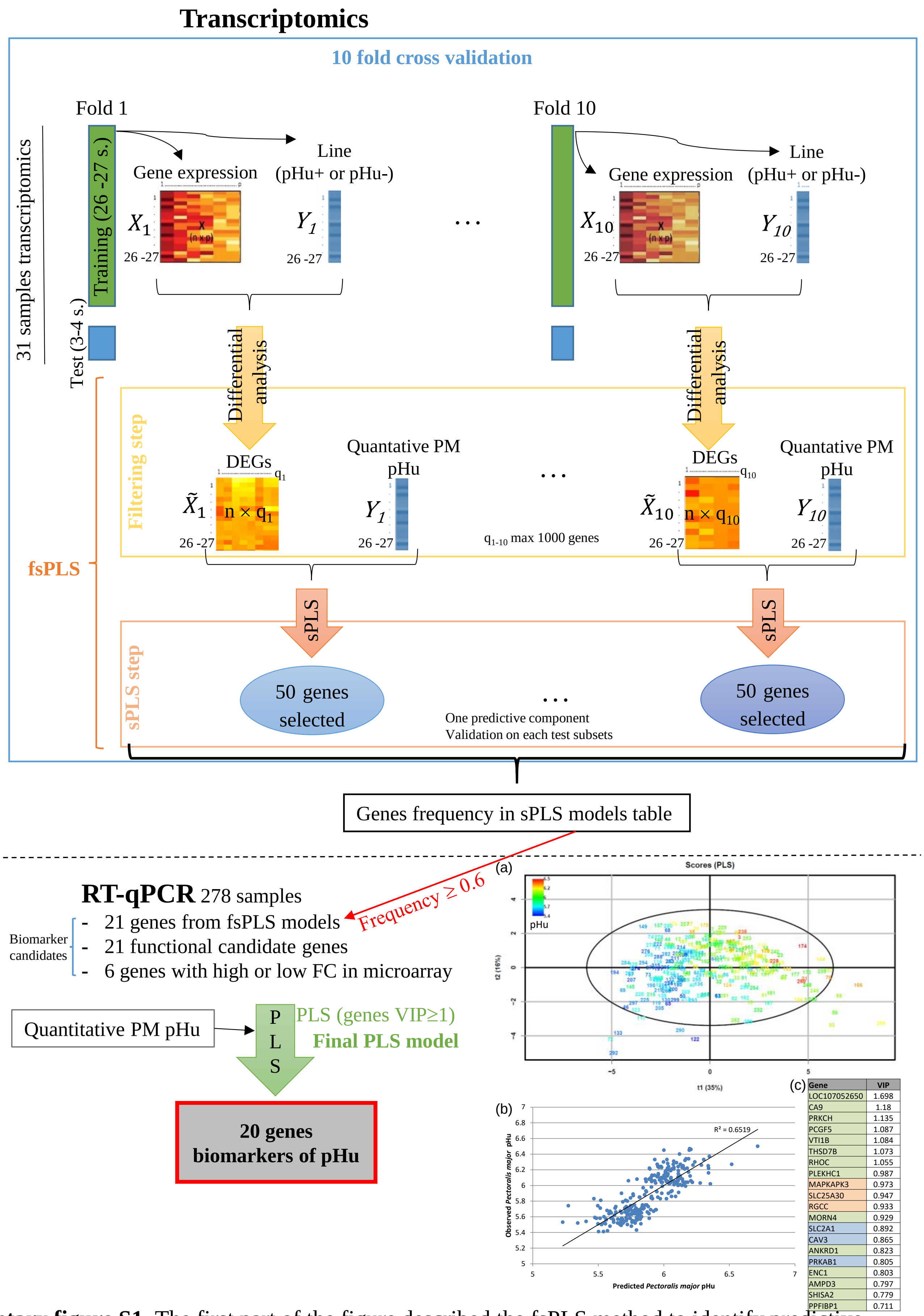

Gene	VIP
LOC107052650	1.698
CA9	1.18
PRKCH	1.135
PCGF5	1.087
VTI1B	1.084
THSD7B	1.073
RHOC	1.055
PLEKHC1	0.987
MAPKAPK3	0.973
SLC25A30	0.947
RGCC	0.933
MORN4	0.929
SLC2A1	0.892
CAV3	0.865
ANKRD1	0.823
PRKAB1	0.805
ENC1	0.803
AMPD3	0.797
SHISA2	0.779
PPFIBP1	0.711

Supplementary figure S1. The first part of the figure described the fsPLS method to identify predictive biomarkers. The validation of the PLS model with 20 genes on a population of 278 chickens was explained in the second part of the figure.

Gene	ID	Supplementary info	Primer sequence (forward /reverse)
**AMPD3**	ENSGALT00000009084	fsPLS	CCCAAGAACGACATCCCAGTA / AACAAACGGAATGCCACCTT
**ANKRD1**	ENSGALT00000010491	fsPLS	GCACAATCATCTGGACACTGG / CACTGAACTGCTGCTCGGTTA
**ATP2B1**	ENSGALT00000018372	fsPLS	TGGCCTCTGTCCTGATTAGTT / CCATTTTGCCCGAGATAACAGAA
**CA9**	ENSGALT00000034416	fsPLS	GACACACCAGCTCATTTCAT / TGTGTAGATGACCTTGGATTTG
CDAN1	ENSGALT00000014915	fsPLS	TGACTGGCTCGTCTCTGAAC / TTGAAATTGAGCTCACAGTGAAAAA
**COG5**	ENSGALT00000039462	fsPLS	CCACAGCTCTACTTCAGGTTAAT / TTCTAATAGCACGTAACAAGGGT
**DYNC1H1**	ENSGALT00000037512	fsPLS	TGAAGTGCTTGCCAATAAGATAGT / TCTTTATCTTCTGAATCCTCTCCCT
**ENC1**	ENSGALT00000024082	fsPLS	ATCGGCTATCAACTGGGTCA / TGTAGGATCTTTAACTTGCATCGT
**HLF**	ENSGALT00000004832	fsPLS	CTCCCAGGCTCAGTTCAGTT / TAGCAGAGGTCGGTGAATTGT
ID3	ENSGALT00000044128	fsPLS	GGGCTCCAATACTCAGACGA / AAAGAAAGCCGGGATTTGGG
**LOC107052650**	XR_001465270	fsPLS	AGCAGAATAAGCAGCGGAGG / AACAGAGACACTAACAGAGAAAGGG
LSMEM1	ENSGALT00000030698	fsPLS	TGAAATTCAGCATCACTCTATTCA / TCACATCAAACAGAATCAAATATCC
**MORN4**	ENSGALT00000009932	fsPLS	CAAAGGCGGACGTGTGTATG / AGCAGACTTGGAGGCAATCT
**MYLIP**	ENSGALT00000020742	fsPLS	GAAGTTTATGACCATGCAAGAAGAG / TTTGTTGGCAGCTAAGACCC
**PCGF5**	ENSGALT00000010507	fsPLS	ATCTGCCTCGACTGTTTACG / ATTGCATAGTACATCCAGCTCAT
**PLEKHC1**	ENSGALT00000020309	fsPLS	TTCCGACATCTGCAAAACCTTC / TGACGGGGCCGATGTAAAGAAT
**PPFIBP1**	ENSGALT00000022836	fsPLS	TTGCTCGTTTCATCACTGCC / ACATCTCTGGTCTACCCCCAAC
**PRKCH**	ENSGALT00000019399	fsPLS	GCTGTATGGCCCTGATGTTG / TGAACGCTTTGAGGATTGCC
**RHOC**	ENSGALT00000002385	fsPLS	GGTCTACGTGCCAACTGTGT / CAGGGATGTTCTCGAGGCTG
**THSD7B**	ENSGALT00000020191	fsPLS	AGTAGCGAGTTGGAATGGCTGA / AGCGCCCATCTATCTCCTCAA
**VTI1B**	ENSGALT00000015509	fsPLS	AGCAGATTGGTGAATACAAGTGA / ACAAAGTCACAAAGGCTCATCT
GADL1	ENSGALT00000018667	High LFC	AGGAGAGAGTGAACAGAGCG / TGCCAAAATTCAGGTCCATCC
**MAPKAPK3**	ENSGALT00000003585	High LFC	ACTCGAACACTGGACAAGCT / GTCATCCTCTCTGTTGGGTCA
**RGCC**	ENSGALT00000027403	High LFC	CCATCAGTAACTCCTTGTAAAGC / AACAAAGTTATCAGACAAAGGCA
RHOBTB3	ENSGALT00000023647	High LFC	TGAGCAGGTTTCAGAGGGTT / GGTCTTAGTGCTAGGTTGGGA
**SHISA2**	ENSGALT00000043654	High LFC	TTTACAACGGAAGGAAAAGCGA / TTAAATCGAGCGCCCTTTCAG
SLC25A30	ENSGALT00000027432	High LFC	GGCATTCTTTCGGGAGTCATT / CTGTGCTGTCAGGGAAACTC
AGL	ENSGALT00000008683	Metabolism / glycogenolysis	TTTGATCGCCACAAGTTCAG / ACTATGTAACCTCCACCACTTT
ALDH7A1	ENSGALT00000013392	Metabolism / Biosynthesis of amino acids	AGATTGGTGGTGCTTTTGGT / CCTGGCACAGTCACTCATAA
AMPD1	ENSGALT00000003247	Metabolism / Purine metabolism	CTTTGTTCCTGTGTTTGAGGCA / CTGCTCTGGCTTTGGACTTTTT
**CAPN2**	ENSGALT00000015245	Metabolism / Protease	ACATCATCGTGCCCTCTACC / GAGATCTCTGCATCGCTTCC
CAPN3	ENSGALT00000032636	Metabolism / Protease	CAAACCAGTGCTCATTCCCT / GCCTGACCCACACTGATTTT
**CAV3**	ENSGALT00000013599	Metabolism / Endocytosis	GAAAGGCAGCTACACCACCT / GTAGATGCGGCTGACACACT
ENO3	NM_205119	Metabolism / Glucose metabolism	CTGTGCACCGGGCAGATTA / GCTGCCCTTATTTGGCTTTG
HIBADH	ENSGALT00000018085	Metabolism / Metabolism of amino acids	CTTTCATGGTTGGTGGAATGGA / TTTTGGCCAGCAGCTTTGG
LDHA	ENSGALT00000039415	Metabolism / Glycolysis and gluconeogenesis	TTAACTTGGTCCAACGCAACGTCAAT / TCCACTGGGTTTGAGACAATCAG
**PDE3B**	ENSGALT00000039611	Metabolism / Insulin signalling pathway	CACCATCTCAGCGAAAATCACA / TCTCCTAGCTGCCGCTCATCTT
PFKFB4	ENSGALT00000003276	Metabolism / Glucose metabolism	GGCAGGAGAACGTCTTGGTT / AGATGGACTCCACTTCGCAC
PGAM1	ENSGALT00000039466	Metabolism / Glucose metabolism	AGGGCAAGAGAGTCCTTATCG / ATGGGCTTCAGGTTCTTGTC
PHKB	ENSGALT00000042911	Metabolism / Glycogen Metabolism	GCTTAACCGACGACAAATAGATGG / CGTCATATCCGATAAGGTTGGTTG

PPP1R3A	ENSGALT00000038901	Metabolism / Glycogen Metabolism	GGCAAACAACGACGACAAAAAC / TTCTTCCTTGCTGCTTGGTGC
**PRKAB1**	ENSGALT00000011925	Metabolism / Glycogen Metabolism	TAAAACCCCCACTCAAGCTCGACC / CCACTGCCCATCCACAAAGAAC
PRKAB2	ENSGALT00000002567	Metabolism / Glycogen Metabolism	CACGTCATGCTCAATCACCTCTAC / CAGACAAAGTGGTGTTTCGTGTCC
PRKAG3	ENSGALT00000038233	Metabolism / Glycogen Metabolism	CCGACAACAATTTCCAGAGCC / TCTGCATCTTGCTGTCCCACAG
**SLC2A1**	ENSGALT00000007808	Metabolism / Glucose transport	ACAACACCGGCGTCATCAA / TTGACATCAGCATGGAGTTACG
SLC2A12	ENSGALT00000022653	Metabolism / Glucose transport	AGAGAGTGGGGAGGTTCCC / TCAGGACGAGCCAAGACA
**SLC2A3**	ENSGALT00000037225	Metabolism / Glucose transport	TGCTCATCTTCTTCATATTCACAT / ACTTCTTTGTCAGGTTCTATGC
SOD1	ENSGALT00000036785	Metabolism / Cellular responses to stress	GATTCTGTCATTTCTCTTACCG / CTCATTTCCCACTGCCATCT
CDK6	ENSGALT00000015428	Reference	TCCAGACCCGCACAACCTATT / TCACCTCACTGGAGTTTTGGC

Supplementary table S4. Primer sequences for the 49 genes analysed by RT-qPCR on male and female population of 278 chickens issued from the pHu lines. The genes highlighted in bold were differentially expressed between pHu+ and pHu- lines using F-test in analysis of variance (p-value ≤ 0.05).

The supplementary information column mention the reason of the selection of genes for validation: the gene issued of the fsPLS model are highlighted in green, those with extreme fold-change based on microarray analysis in orange, and those whose function is directly related to carbohydrate and muscle metabolism in blue.
